# Effects of different interventions on animal models of ischemic stroke: Protocol for an overview and a network meta-analysis

**DOI:** 10.1097/MD.0000000000015384

**Published:** 2019-04-26

**Authors:** Yan Zhang, Hui-Jin Yu, Shu-Zhen Shi, Jian-Cheng Wang

**Affiliations:** a Spinal Cord Injury Rehabilitation Department, Rehabilitation Center Hospital of Gansu Province; b School of Basic Medical Sciences, Lanzhou University; c Gansu Provincial Hospital, Lanzhou City, Gansu Province, China.

**Keywords:** animal models, ischemic stroke, network meta-analysis, overview

## Abstract

**Background:**

Ischemic stroke often leads to lifelong disability or death in stroke patients. It is one of the most common causes of death and disability worldwide, so it is a global health problem. The objective of this protocol is to provide the methods for using overview and network meta-analysis to identify the more effective intervention for infarct volume and neurobehavioral score in animal models of ischemic stroke.

**Methods:**

A systematic literature search will be conducted in PubMed and Embase to obtain relevant systematic reviews on December 11, 2018. Assessing the Methodological Quality of Systematic Reviews (AMSTAR2) and SYRCLE's risk of bias tool will be used to assess quality of the included reviews and risk of bias of randomized controlled trials (RCTs) for animal studies. Infarct volume and neurobehavioral score will be chosen as primary and secondary outcomes. The relative effect size of the treatment will be calculated using the standardized mean difference (SMD) and 95% confidence interval (CI). R 3.5.1 through the GEMTC package will be used to perform a network meta-analysis to synthesize direct and indirect evidence.

**Results:**

The results of this paper will be submitted to a peer-reviewed journal for publication.

**Conclusion:**

Our study can provide a reference for further clinical practice and can be compared with clinical trial results to obtain a more credible therapeutic effect of this intervention.

**Ethics and communication:**

Formal ethical approval is unnecessary, because this study is based on published researches.

**PROSPERO registration number:**

CRD42019126811

## Abstract 2

Supplemental Digital Content is available in the text

## 1 Introduction

Ischemic stroke, also known as cerebrovascular accident, is caused by the decreased or interrupted blood supply in part of the brain,^[1]^ it remains one of most common causes of death and disability worldwide,^[2]^ is a main global health concern that often leads to lifelong disability or death of patients.^[3]^ Yet, preventive strategies thus far have been relatively ineffective in curbing the global stroke burden.^[4]^ Stroke patients and their families incur steep social and medical burdens.^[5]^ Over 80% of stroke events are ischemic and correlate with traditional cardiovascular risk factors—namely, age, male sex, the presence of hypertension, diabetes mellitus, atrial fibrillation, and dyslipidemia.^[6]^ Ischemic strokes have been further categorized into subtypes according to the mechanism of injury. And ischemic stroke can be classified, according to etiology as: large vessel atherosclerosis, cardioembolic, small vessel atherosclerosis (lacunes), other determined etiology, or undetermined etiology.^[7]^ The majority, ∼60%, of all new ischemic strokes are classified as large-artery atherosclerosis, cardioembolic, or small vessel diseases.^[8]^ Behavioral impairment, cognitive impairment, and mood impairment are all problems faced by stroke survivors. Among them, post-stroke depression has been recognized by psychiatrists for more than 100 years, but it was not until the 1970s that relevant controlled systematic studies appeared.^[9]^ Studies have shown that even mild strokes can increase the risk of cognitive impairment in survivors and affect their quality of life.^[10]^

At present, many studies have reported on the different intervention to improve the functional and structural prognosis of ischemic stroke. In general, certain treatments are preceded by animal experiments prior to clinical application. However, although for the same intervention, there may be some bias due to different implementers, subjects, and other factors. Therefore, there has been a second study of animal studies. An influential commentary which published in the Lancet (2002),^[11]^ first clarify the scientific rationale for systematic reviews (SRs) of animal studies, awareness of the value of SRs in experimental animal research has steadily increased.^[12]^ Currently, many studies have be performed to obtain more reliable results after the secondary analysis of these same kind of original studies through SRs or meta-analysis.^[13–16]^ From these trials or SRs, we cannot know which intervention is better, Therefore, In this study, we collect relevant SRs through retrieval to reanalyze the results of these SRs and to obtain the included randomized controlled trials (RCTs) to conduct network meta-analysis (NMA) or indirect comparative analysis on all interventional RCTs in animal models of ischemic stroke hoping to obtain the optimal intervention, that can provide a reference for clinical practice and can be compared with clinical trial results to obtain a more credible therapeutic effect of this intervention.

## 2 Objectives

This overview and NMA aim to evaluate the evidence for the effectiveness of different interventions in ischemic stroke of animal models.

## 3 Methods and analysis

Studies^[17,18]^ have mentioned that register in advance can improve the methodological quality of systematic reviews. This review has been registered on the International Prospective Register of SRs (PROSPERO), registration number: CRD42019126811. (http://www.crd.york.ac.uk/PROSPERO/display_record.php?ID=CRD42019126811).

This is an overview of SRs and NMA of RCTs, so ethical approval is not necessary.

### 3.1 Eligibility criteria

Eligibility criteria have been prepared in terms of the participants (P), intervention (I), comparator (C), outcomes (O), and study design (S). The specific inclusion and exclusion criteria are as follows:

#### 3.1.1 Inclusion criteria

a. Participants
  Animals with ischemic stroke. No restriction of species and sexes.
b. Intervention
  Treated with: drugs, such as Granulocyte-Colony Stimulating Factor, statins; other treatments: such as therapeutic hypothermia, acupuncture, different training strategies.
c. Comparator
  The control group treated with other drugs, or other training strategies, or placebo, or sham treated, or no treatment.
d. Outcomes
  We will choose infarct volume as our primary outcome and neurobehavioral score as our secondary outcome.
e. Study design
  To be included, SRs must include controlled trials with different interventions; include the results of meta-analysis; and satisfy the participants, interventions, controls and outcomes of interest criteria described.

Each of the above 5 items is required.

#### 3.1.2 Exclusion criteria

a. Participants
  Animals with co-morbidities, for example, with other head diseases besides ischemic stroke, or diseases related to the treatment effect, or ex vivo studies, or in vitro studies, or studies in humans or in silico studies.
b. Intervention
  The specification and usage of the medicine were not specified
c. Comparator
  The specification and usage of the medicine were not specified.
d. Outcomes
  Outcomes without infarct volume and neurobehavioral score.
e. Study design
  Case studies, cross-over studies, studies without a separate control group, primary studies, SRs which only reported data narratively.
f. Duplication
  Duplicate records are excluded. For repeated studies that have been updated, the older one will be excluded, or can be used as supplementary data in further research.

If 1 of the above 6 criteria is met, the citation is excluded

### 3.2 Literature search and selection

Two international databases PubMed and Embase were searched for relevant SRs published in English from inception to December 11, 2018. The search strategy combines medical subject headings (MeSH) and free words. The search terms in the search strategy are mainly based on research objects and research design. The research object: animal model and stroke, the research type is mainly meta-analysis or SR, their synonyms or related words respectively collected, and then use the logical operators “OR” and “AND” to form a complete search strategy (Appendix 1).

**Figure 1:**
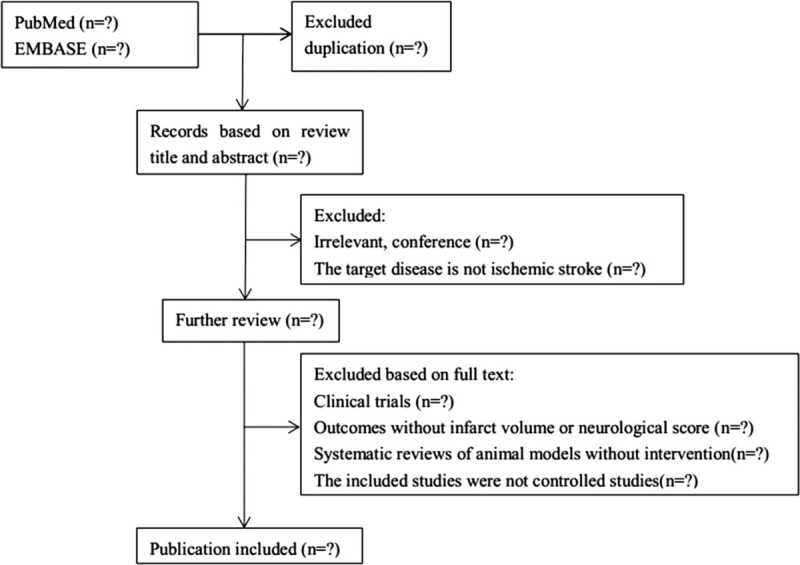
PRISMA flow diagram of including studies.

Literature search results will be imported into ENDNOTE X8 software. The duplicates will be removed, 2 independent reviewers will examine the title and abstract of retrieved records by inclusion criteria to identify relevant SRs/meta-analyses and then the same 2 authors will examine full text according to the eligibility criteria independently. At the same time, RCTs included in the SRs that meet the inclusion criteria and similar RCTs in the reference will be collected to conduct NMA. Any disagreement will be resolved by the discussion between the two reviewers, or through arbitration by third party. A flow diagram will be presented to describe the process of study selection (Fig. 1).

### 3.3 Data collection and analysis

#### 3.3.1 Data collection

Two reviewers will establish a form using Microsoft Excel 2010, pilot and refine this form using 3 initial studies. After the form has been developed, the 2 reviewers will extract data from the text and figure/table independently. First, information of included reviews will be extracted as follows: title, the first author, the name of the journal, year of publication, PICOS, numbers of RCTs included in each SR, summary effect estimates for main outcomes, overall risk of bias, publication bias, and conclusions. Then, for duplicate citations of each eligible obtained randomized controlled trial, the recently updated RCTs will be selected for data extraction, while the older versions will be used as supplementary information, if necessary. The following data will be extracted from each embedded RCTs: therapy method of interventions, comparators, number of participants, outcomes, and other study information, including title, country, treatment duration, and outcomes(the 2 outcome indexes, infarct volume and neurobehavioral score which we extracted are continuous variables, they will be presented on mean value and standard deviation).

#### 3.3.2 Data analysis

a. Basic characteristics
  We will provide a comprehensive description of the basic characteristics for the included SRs.
b. NMA of included RCTs

In the absence of direct comparisons of all interventions, indirect comparative analysis of NMAs using different RCTs can provide useful evidence for health care decisions,^[19]^ so NMA will be conducted on both direct evidence and indirect evidence. Because of the exploratory nature of animal study, a random effects model will be used. *I*^2^ statistic will be calculated for quantifying heterogeneity among included RCTs. The *I*^2^ statistic of <25%, 26% to 50%, are regarded as low, moderate heterogeneity, respectively. Where *I*^2^ statistic of 50% or more indicated a considerable heterogeneity. If the heterogeneity is significant, we will further analyze whether it is clinical or methodological. In order to avoid the impact of excessive heterogeneity, it is possible to conduct descriptive analysis instead of data synthesis. A consistency model will be drawn for each evaluated outcome and the relative effect size of the treatment will be calculated using the standardized mean difference (SMD) 95% confidence interval (CI) for the continuous variables. Node splitting method will be used to examine the inconsistency between direct and indirect comparisons if a loop connecting 3 or more arms exist. If node-splitting analysis determined *P* > .05, the consistency model will be used for pooled analysis. Otherwise, the inconsistency model will be used. Additionally, the convergence will be assessed using the potential scale reduction factor (PSRF) and the Brooks–Gelman–Rubin (BGR) method, and a value of 1 indicates a good convergence. The analyses will be performed using R 3.5.1.

### 3.4 Quality assessment/methodological quality of included reviews

The methodological quality of a systematic review reflects risk of bias or validity in its process and results.^[20]^ In this paper, quality assessment of the included reviews, and risk of bias of the RCTs for animal studies conducted by 2 trained authors independently according to Assessing the Methodological Quality of Systematic Reviews (AMSTAR2)^[21]^ and SYRCLE's risk of bias tool,^[22]^ respectively. Discrepancies were resolved after discussion between the 2 authors or were referred to an arbitrator.

AMSTAR2 is usually used to assess the degree to which review methods avoided bias by evaluating the methods against 16 distinct criteria.^[23]^ Therefore, we will use AMSTAR2 to assess the methodological quality of all SRs included in this overview. AMSTAR2 contains a total of 16 items, in our study, each item has 4 ratings, respectively “Yes,” “Partial yes” and ”No, Not applicable,“ corresponding to ”1,“ ”0.5“ and ”0,“ so the total score is ranging from 0 to 16. According to the score, the quality is divided into 4 grades, 0 to 3 is critically low, 4 to 7 is low, 8 to 11 is moderate, and 12 to 16 is high. The assessment shall be made by 2 reviewers independently. If there is any disagreement, it will be resolved by discussion. If there still no consensus can be reached, then judged by the third experienced reviewer.

SYRCLE's risk of bias tool will be used to assess the risk of included RCTs in NMA. SYRCLE's risk of bias tool consists of a domain-based instrument with 10 items related to 6 types of bias: selection bias, performance bias, detection bias, attrition bias, reporting bias and other biases. These 10 items are organized in subitems in the form of questions that support a “Yes,” “No,” “Unclear answer.” ”Yes“ refers to low bias with low risk; ”No“ refers to high bias with high risk; ”unclear" is the degree of risk is uncertain. Finally, the evaluation results of all the included original literatures were displayed by text, table or figure.

### 3.5 Dealing with missing data

If the data needed is missing or incomplete, we will contact the corresponding author or the first author by email for relevant information. If there is no response, this record is excluded.

### 3.6 Sensitivity analysis

If necessary, the effect of each study on the random effects model will be assessed using sensitivity analysis. The exclusion method was used to analyze the sensitivity of the overall combined effect of all outcome indicators, that is, each study will be excluded, and the remaining studies were re-analyzed to determine the stability of the results. If the results show that there is no qualitative change in the combined effect, the results are stable.

### 3.7 Publication bias

If there are 10 or more studies in the NMA, we will use the funnel plot to evaluate the potential publication bias. Descriptive analysis is made by the symmetry of funnel plot, the graph is asymmetrical and does not show inverted funnel shape, suggesting that there may be publication bias. It is possible related to that the literature with negative results is not easy to publish and the quality of the included literature method is low. And quantitative analysis is made on publication bias by the method of Egger's test or Begg's test.

### 3.8 Subgroup analysis

If necessary, we will perform subgroup analysis on different animal species.

## 4 Discussions

Drugs are usually tested for the effectiveness and safety in animal models before clinical trials. This is due to the high cost of large-scale clinical trials and risks that cannot prove clinical utility, so animal models play an important role.^[24]^ The purpose of the animal being used for research has been controversial, mainly considering ethical issues of whether humans have the right to use of animals. Therefore, animal experiments are considered acceptable only when the benefits of the proposed experiment are outweigh the suffering of the animal and there is no alternative.^[25]^ A study^[26]^ comparing the effects of animal experiments with clinical trials mentioned that the animal model of stroke consistent with the results of clinical trials seems to be more representative of human conditions than animal models of brain injury that differ in outcomes, so we suspect that animal models of stroke may be more easily replicated and that the experimental results may be more consistent with clinical results. However, some studies have shown that the positive results can be amplified several times if randomization and blindness are not used in animal experiments.^[27]^ So the experimental results of animal models have a great contribution to clinical practice, but due to the natural gap between animals and people, the results are not necessarily consistent with clinical trials. Furthermore, due to the some bias, such as implementation bias in animal model experiment can also to a certain extent, affect the authenticity of the test results. Even so, it still makes sense to conduct a comprehensive analysis of the experimental results of animal models, at least to find out the most effective interventions in animal models of cerebral ischemia, and provide clues for clinical research, so as to further explore the evidence for the effectiveness in humans.

Taken together, the therapeutic measures that we are going to find in this article to beneficial the infarct volume and neurological score of ischemic stroke still need to be interpreted cautiously in clinical application.

## Author contributions

**Data curation:** Yan Zhang, Hui-Jin Yu.

**Formal analysis:** Hui-Jin Yu, Shu-Zhen Shi.

**Project administration:** Jian-Cheng Wang.

**Writing – original draft:** Yan Zhang, Shu-Zhen Shi.

## Supplementary Material

Supplemental Digital Content
