# Supplementary material for: Effects of different interventions on animal models of ischemic stroke: Protocol for an overview and a network meta-analysis
Source: Medicine (Baltimore). 2019 Apr 26;98(17):e15384. doi: 10.1097/MD.0000000000015384 (PMC6831226; doi:10.1097/MD.0000000000015384)
Supplement: Supplemental Digital Content [file medi-98-e15384-s001.docx]

PubMed

#1 "Meta-Analysis as Topic"[Mesh] OR "Meta-Analysis"[Publication Type]

#2 meta analysis[Title/Abstract] OR meta analyses[Title/Abstract] OR metaanalysis[Title/Abstract] OR metanalysis[Title/Abstract] OR met-analysis[Title/Abstract] OR metaanalyses[Title/Abstract] OR metanalyses[Title/Abstract] OR met-analyses[Title/Abstract] OR systematic review[Title/Abstract] OR systematic reviews[Title/Abstract]

#3 #1 OR #2

#4 "Models, Animal"[Mesh]

#5 animal disease model*[Title/Abstract] OR animal model*[Title/Abstract] OR avian model*[Title/Abstract] OR chicken model*[Title/Abstract] OR poultry model*[Title/Abstract] OR bovine model*[Title/Abstract] OR cattle model*[Title/Abstract] OR cow model*[Title/Abstract] OR canine model*[Title/Abstract] OR dog model*[Title/Abstract] OR caprine model*[Title/Abstract] OR goat model*[Title/Abstract] OR equine model*[Title/Abstract] OR horse model*[Title/Abstract] OR feline model*[Title/Abstract] OR cat model*[Title/Abstract] OR fish model*[Title/Abstract] OR piscine model*[Title/Abstract] OR frog model*[Title/Abstract] OR fruit fly model*[Title/Abstract] OR drosophila model*[Title/Abstract] OR invertebrate model*[Title/Abstract] OR nematode model*[Title/Abstract] OR ovine model*[Title/Abstract] OR sheep model*[Title/Abstract] OR porcine model*[Title/Abstract] OR pig model*[Title/Abstract] OR swine model*[Title/Abstract] OR primate model*[Title/Abstract] OR monkey model*[Title/Abstract] OR simian model*[Title/Abstract] OR macaque model*[Title/Abstract] OR rabbit model*[Title/Abstract] OR leporine model*[Title/Abstract] OR rodent model*[Title/Abstract] OR chinchilla model*[Title/Abstract] OR gerbil model*[Title/Abstract] OR experimental gerbil[Title/Abstract] OR jird model*[Title/Abstract] OR laboratory gerbil[Title/Abstract] OR guinea pig model*[Title/Abstract] OR hamster model*[Title/Abstract] OR murine model*[Title/Abstract] OR murin model*[Title/Abstract] OR mouse model*[Title/Abstract] OR rat model*[Title/Abstract]

#6 #4 OR #5

#7 "Stroke"[Mesh] OR "Brain Infarction"[Mesh] OR "Brain Stem Infarctions"[Mesh] OR "Lateral Medullary Syndrome"[Mesh] OR "Cerebral Infarction"[Mesh] OR "Dementia, Multi-Infarct"[Mesh] OR "Infarction, Anterior Cerebral Artery"[Mesh] OR

"Infarction, Middle Cerebral Artery"[Mesh] OR "Infarction, Posterior Cerebral Artery"[Mesh] OR "Stroke, Lacunar"[Mesh]

#8 ACA Infarction*[Title/Abstract] OR Acute Cerebrovascular Accident[Title/Abstract] OR acute cerebrovascular lesion[Title/Abstract] OR acute focal cerebral vasculopathy[Title/Abstract] OR Anterior Cerebral Artery Infarction*[Title/Abstract] OR Anterior Cerebral Artery Syndrome[Title/Abstract] OR Anterior Cerebral Circulation Infarction*[Title/Abstract] OR apoplexia[Title/Abstract] OR apoplexy[Title/Abstract] OR Benedict Syndrome[Title/Abstract] OR brain accident[Title/Abstract] OR brain attack[Title/Abstract] OR Brain Infarction*[Title/Abstract] OR brain insult[Title/Abstract] OR brain insultus[Title/Abstract] OR brain ischaemic attack[Title/Abstract] OR brain ischemic attack[Title/Abstract] OR Brain Stem Infarction*[Title/Abstract] OR brain vascular accident[Title/Abstract] OR Brain Venous Infarction*[Title/Abstract] OR brainblood flow disturbance[Title/Abstract] OR Brainstem Infarction*[Title/Abstract] OR cerebral apoplexia[Title/Abstract] OR Cerebral Infarction*[Title/Abstract] OR cerebral insult[Title/Abstract] OR cerebral vascular accident[Title/Abstract] OR cerebral vascular insufficiency[Title/Abstract] OR cerebro vascular accident[Title/Abstract] OR cerebrovascular accident[Title/Abstract] OR Cerebrovascular Apoplexy[Title/Abstract] OR cerebrovascular arrest[Title/Abstract] OR cerebrovascular failure[Title/Abstract] OR cerebrovascular injury[Title/Abstract] OR cerebrovascular insufficiency[Title/Abstract] OR cerebrovascular insult[Title/Abstract] OR cerebrum vascular accident[Title/Abstract] OR Claude Syndrome*[Title/Abstract] OR Dementia Multi Infarct*[Title/Abstract] OR Dementia Multi-Infarct*[Title/Abstract] OR Foville Syndrome*[Title/Abstract] OR Heubner Artery Infarction*[Title/Abstract] OR Heubners Artery Infarction*[Title/Abstract] OR Heubner's Artery Infarction*[Title/Abstract] OR ischaemic cerebral attack[Title/Abstract] OR ischaemic seizure[Title/Abstract] OR ischemic cerebral attack[Title/Abstract] OR ischemic seizure[Title/Abstract] OR Lacunar Dementia*[Title/Abstract] OR Lacunar Infarct*[Title/Abstract] OR lacunar infarction*[Title/Abstract] OR Lacunar Syndrome*[Title/Abstract] OR Lateral Medullary Syndrome*[Title/Abstract] OR MCA Infarction[Title/Abstract] OR Middle Cerebral Artery Circulation Infarction*[Title/Abstract] OR Middle Cerebral Artery Embolic Infarction*[Title/Abstract] OR Middle Cerebral Artery Infarction*[Title/Abstract] OR Middle Cerebral Artery Syndrome*[Title/Abstract] OR Middle Cerebral Artery Thrombosis[Title/Abstract] OR Middle Cerebral Artery Thrombotic Infarction*[Title/Abstract] OR Millard Gublar Syndrome*[Title/Abstract] OR Millard-Gublar Syndrome*[Title/Abstract] OR Multi Infarct Dementia*[Title/Abstract] OR Multiinfarct Dementia*[Title/Abstract] OR Multi-Infarct Dementia*[Title/Abstract] OR PCA Infarction*[Title/Abstract] OR Posterior Cerebral Artery Embolic Infarction*[Title/Abstract] OR Posterior Cerebral Artery Infarction*[Title/Abstract] OR Posterior Cerebral Artery Syndrome*[Title/Abstract] OR Posterior Cerebral Artery Thrombotic Infarction*[Title/Abstract] OR Posterior Circulation Brain Infarction*[Title/Abstract] OR Stroke[Title/Abstract] OR Subcortical Infarction*[Title/Abstract] OR Venous Brain Infarction*[Title/Abstract] OR Weber Syndrome*[Title/Abstract]

#9 #7 OR #8

#10 #3 AND #6 AND #9
